# Luminescence Thermometry Probes Local Heat Effects at the Platinum Electrode Surface during Alkaline Water Electrolysis

**DOI:** 10.1021/acsenergylett.4c01238

**Published:** 2024-06-13

**Authors:** Thimo S. Jacobs, Sunghak Park, Marco Schönig, Bert M. Weckhuysen, Marc T.M. Koper, Ward van der Stam

**Affiliations:** † Inorganic Chemistry and Catalysis, Debye Institute for Nanomaterials Science & Institute for Sustainable and Circular Chemistry, Utrecht University, 3584 CG Utrecht, The Netherlands; ‡ Leiden Institute of Chemistry, Leiden University, 2300 RA Leiden, The Netherlands; ∥ SKKU Institute of Energy Science and Technology (SIEST), Sungkyunkwan University, Suwon 16419, Republic of Korea

## Abstract

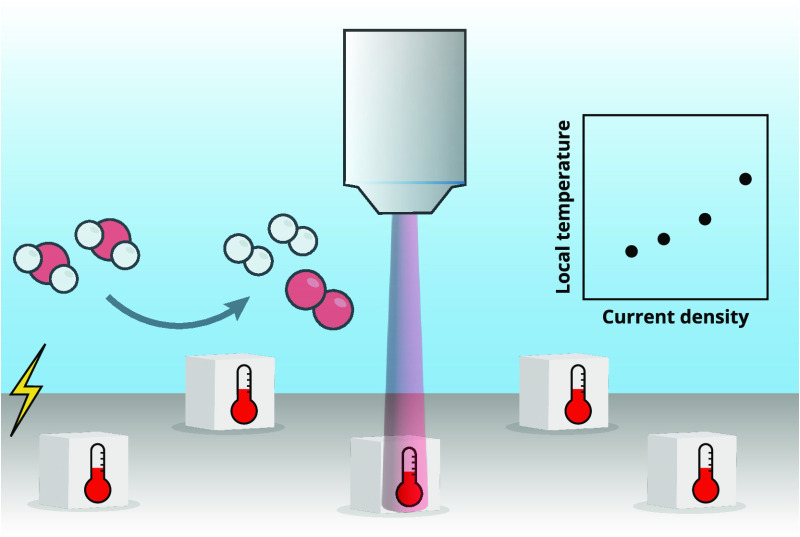

Accurate determination of the temperature dynamics at the electrode surface is crucial for advancing electrocatalysis, particularly in the development of stable materials that aid energy conversion and storage technologies. Here, lanthanide-based *in situ* luminescence thermometry was used to probe local heat effects at the platinum electrode surface during alkaline water electrolysis. It is demonstrated that the oxygen evolution reaction (OER) induces a more significant temperature increase compared to the hydrogen evolution reaction (HER) under the same electrochemical conditions. This difference is attributed to variations in overpotential heating and local effects on Joule heating. Furthermore, local heat effects are not observed at increased electrolyte concentrations during the HER, whereas substantial temperature variations (up to 2 K) are detected for the OER at higher electrolyte concentrations. Our observations highlight the potential of *in situ* luminescence thermometry to measure interfacial temperature effects during electrocatalytic reactions.

Temperature is a fundamental parameter that significantly influences the thermodynamics, kinetics, and overall efficiency of (electro)chemical processes.^1−4^ For example, the equilibrium electrode potential directly depends on the temperature via the Nernst equation.^5^ Additionally, driving nonspontaneous or irreversible electrochemical reactions (*e*.*g*., alkaline water electrolysis) leads to the evolution of heat and thus a temperature change directly at the surface, which can result in severe (local) heating of the electrode or electrochemical cell. These local heat effects can influence the structure of the electrode material and hence drastically impact the overall process or can alter the reaction kinetics. Thus, precise determination and understanding of local temperature effects and dynamics through detailed *in situ* or *operando* experiments are imperative for the design and optimization of catalysts and reaction conditions.^6,7^ State-of-the-art temperature measurements under electrochemical conditions are typically conducted at the backside of the working electrode using a thin electrode-temperature sensor assembly with, *e*.*g*., pyroelectric sensors,^8^ thermocells,^9^ thermistors,^10,11^ or heat flux sensors,^12^ which allow for sensitive detection of temperature changes up to 10^–5^ K, necessary to obtain thermodynamic information on adsorbed intermediates.^13^ In a recent study, infrared thermography allowed for spatially resolved temperature measurements by probing the backside of the electrode in a gas diffusion configuration.^14^ To the best of our knowledge, direct and local temperature measurements at or near the electrode surface during an electrochemical process have not yet been performed.

A promising candidate to measure the temperature directly at the surface of the electrode is luminescence thermometry, which has already been used to measure the local catalyst temperature during thermocatalytic reactions.^15−17^ For example, temperature-dependent emission from lanthanide-based nano- or microsized probes is collected by ratiometric luminescence thermometry. Typically, these probes are embedded within or attached to the surface of the catalyst and give a local measure for temperature.^15−17^ Other luminescent materials used as probes include rare-earth-doped oxides and quantum dots.^18^ One of the key advantages of luminescence thermometry over other temperature measurements (*e*.*g*., IR camera) is its ability to provide highly localized temperature measurements on the nanometer scale.^19^ By placing these probes closer to the surface of the working electrode, it could allow for studies of temperature gradients and hotspots that may exist within the electrochemical system, offering valuable insights into local temperature effects on electrocatalytic performance.

In this work, we developed a bifunctional electrode that can measure the local temperature at the platinum electrode surface during alkaline water electrolysis (*i*.*e*., hydrogen and oxygen evolution) through luminescence thermometry. This technique was used to study local heat effects during the platinum-catalyzed hydrogen and oxygen evolution reactions, HER and OER, respectively.^20^ Local temperature measurements at different alkaline electrolyte concentrations and at different current densities for both the HER and the OER revealed strong current-dependent trends in heat generation. Larger temperature variations were observed at higher current densities for the OER than for the HER. It was found that an increase in electrolyte concentration effectively mitigated the heat generation effects during the HER, whereas large temperature variations were still observed for the OER at higher electrolyte concentrations. Notably, the temperature increase was consistently larger during the OER compared to the HER under the given conditions, which could be ascribed to greater overpotential heating and/or enhanced Joule heating. This might arise from differences in local conductivities and resistance of electrode and electrolyte as well as different gas bubble fouling effects associated with each reaction. Our measurements highlight the potential of luminescence thermometry to probe local heat effects during electrocatalytic reactions. The potential for spatially resolved local temperature measurements could also help in, for example, the identification of inactive parts of the catalyst. Using these insights into local heat effects, luminescence thermometry could help in the determination and understanding of temperature dynamics at the electrode–electrolyte interface, allowing for a further understanding of the electrocatalyst structure-performance relationship beyond alkaline water electrolysis.

To locally measure the temperature at the electrode surface, we designed an electrode, as depicted in Figure 1a. Fluorine-doped tin oxide (FTO) was chosen as substrate, as it is both transparent and conductive, allowing for collection of the temperature-dependent emission light through the FTO. We opted for an upside-down configuration, as the gas bubble formation during water electrolysis would otherwise severely hinder the collection of the emitted light with the microscope objective due to the scattering. The FTO substrate was first covered with Nd^3+^-doped Y_2_O_3_, which are micrometer-sized particles that act as the temperature probe due to the temperature-dependent emission of Nd^3+^. As the particle size is close to the resolution of the microscopy setup, emitted light from the entire Y_2_O_3_:Nd^3+^ particle will be collected. The measured Δ*T* is therefore a minimum value for the temperature change. Subsequently, a uniform thin layer of platinum, approximately 100 nm in thickness, was deposited onto the Y_2_O_3_/FTO substrate using sputtering. The resulting electrode was annealed at 550 °C for 70 h to enhance mechanical adhesion of the sample while preventing significant agglomeration of the sputtered Pt. As shown in Figures 1b and S2, the surface of the prepared bifunctional electrode was completely covered with Pt. The surface condition of the prepared electrode was evaluated using blank voltammetry measurements in a 0.5 M KOH electrolyte under ambient air conditions (Figure 1c). Despite the downward shift in the overall graph, which is attributed to the reduction of residual dissolved oxygen in the electrolyte, the hydrogen underpotential deposition peaks and Pt oxidation/reduction peaks were clearly distinguishable.^21,22^ This observation in the blank voltammetry is in agreement with the SEM results, confirming the complete coverage of the electrode surface with Pt. Furthermore, the minor difference between scans 1 and 50 during cyclic voltammetry confirms the stability of the bifunctional electrode (Figure 1c).

**Figure 1:**
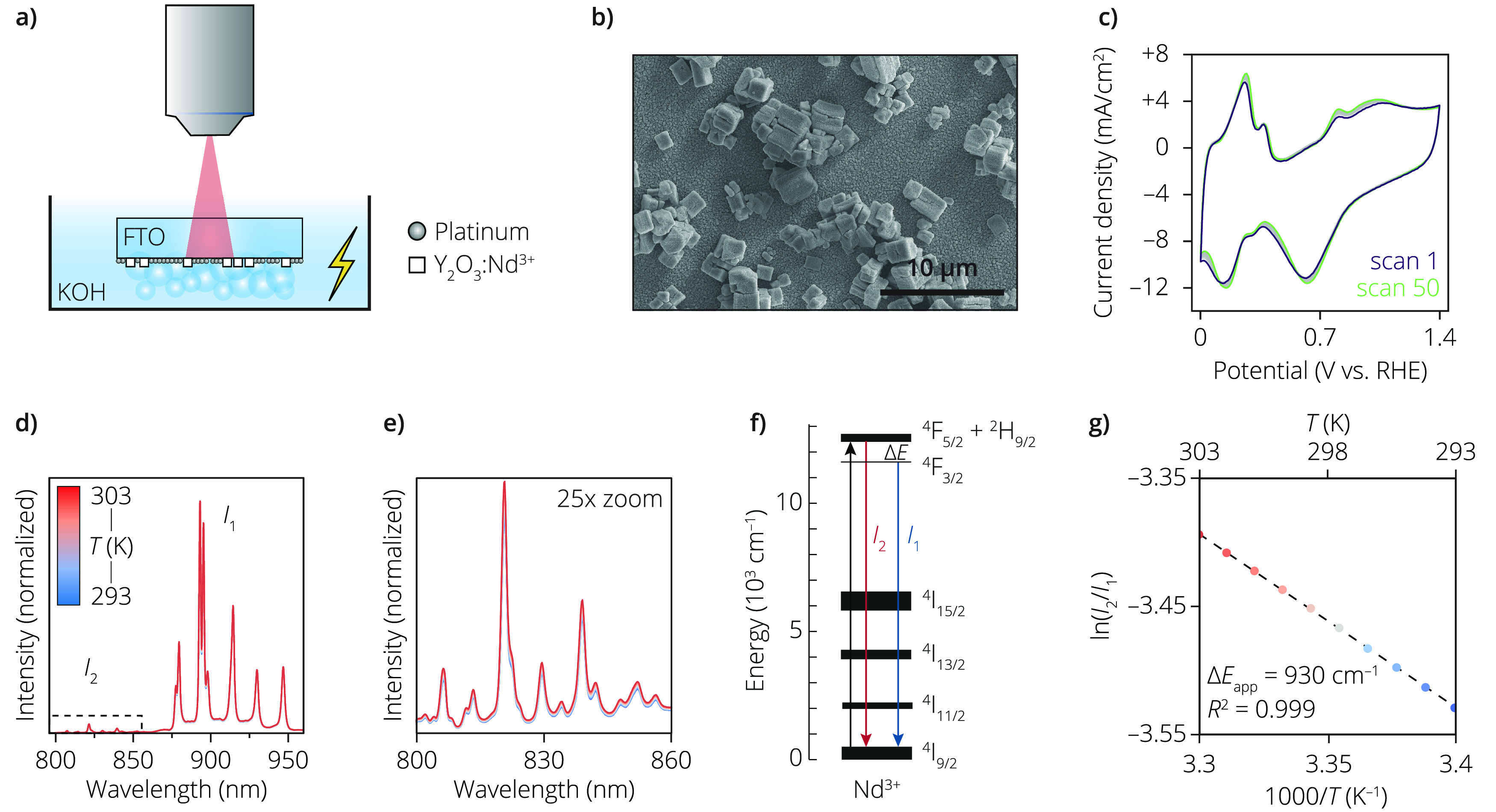
(a) Illustration of the sample configuration for the temperature measurements on the surface of the electrode. The thermometry particles, Y_2_O_3_:Nd^3+^ (see Figure S1 for additional characterization), were placed on top of a fluorine-doped tin oxide (FTO) substrate. A layer of 100 nm platinum was sputtered, resulting in coverage of the FTO and thermometry particles. (b) Scanning electron microscopy (SEM) image of the surface of the electrode, where the larger cubic particles are the thermometry particles (see Figure S2 for additional images on the Pt coverage). The scalebar is 10 μm. (c) Cyclic voltammetry scans 1 and 50 of the bifunctional electrode in 0.5 M KOH, measured from 0 to 1.4 V vs reversible hydrogen electrode (RHE), with a scan speed of 500 mV/s. (d) Spectra of the temperature-dependent emission from microcrystalline Y_2_O_3_ doped with 1.2% of Nd^3+^, collected after 785 nm excitation at temperatures between 293 and 303 K (with steps of 1 K, measured in air). (e) Zoom-in on the *I*_2_ region of the spectrum to illustrate the temperature dependency of the spectra. The same color scale applies as in (d). (f) Energy-level diagram of a single Nd^3+^ ion, with the black arrow representing the 785 nm excitation and the colored arrows marking the temperature-dependent emission of *I*_2_ (red) and *I*_1_ (blue) as also shown in (d), respectively. (g) The natural logarithm of the integrated intensity ratio between *I*_2_ (795–860 nm) and *I*_1_ (860–960 nm) versus 1/*T* for the spectra in (d) and fitted to the Boltzmann model in eq 1 (black dotted line), which is used as calibration curve for *in situ* temperature measurements.

Figure 1d shows the temperature-dependent emission spectra of the Nd^3+^-doped Y_2_O_3_, after excitation with a 785 nm laser source. As controlled heating of the electrolyte is nontrivial in the open-cell configuration that is used for the electrochemical experiments, we performed the calibration in air in a Linkam cell (see Supporting Information for additional details). Figure 1e is a zoom-in on spectral area *I*_2_, showing that with increasing temperature, the emission intensity of *I*_2_ increases compared to *I*_1_. The emission in the two spectral areas of *I*_1_ and *I*_2_, respectively, is originating from two thermally coupled excited state energy levels of Nd^3+^ back to the ground state: from the ^4^F_3/2_ level at 860–960 nm and from the ^4^F_5/2_ level at 795–860 nm. Figure 1f shows the energy-level scheme of Nd^3+^ and the relevant absorption and emission transitions. The natural logarithm of the ratio of *I*_2_/*I*_1_, the so-called luminescence intensity ratio (LIR), scales linearly with the inverse of the temperature (Figure 1g). We can calibrate the thermometer probes by fitting the data in Figure 1g to a Boltzmann model:
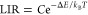
1where *k*_B_ is Boltzmann’s constant, *C* is a unitless prefactor, and Δ*E* is the energy difference between the thermally coupled excited states. The calculated apparent energy difference between the emitting levels of Δ*E*_app_ = 930 cm^–1^ is lower than commonly found in literature (around 990 cm^–1^).^23^ As minor temperature increases are expected during electrocatalytic processes in aqueous electrolytes, the calibration was performed in a small temperature window of 10 K, whereas other reports determine Δ*E* over a much wider temperature window. However, it is evident that the excited-state populations still clearly follow Boltzmann statistics (i.e., linear dependence of LIR with 1/*T*), and hence, we can use the value of Δ*E*_app_ to convert the LIR values to temperatures for the remainder of this work.

We performed the temperature measurements during both hydrogen and oxygen evolution reactions in alkaline conditions in different electrolyte concentrations at varying current densities (up to 150 mA/cm^2^), similar to alkaline water electrolysis reports in literature.^24^ We investigated the hydrogen evolution reaction at the bifunctional working electrode during chronopotentiometry (CP) by applying negative currents ranging from −20 to −150 mA/cm^2^ while measuring the applied potential (Figure S4). Sequential measurements were conducted at different current densities to analyze the impact of current density on the temperature at the electrode–electrolyte interface for the same electrode at the same spot. Figure 2a displays the temperature readouts at each current density, where the collection of emission spectra is initiated at *t* = 0 s. Following the acquisition of several emission spectra, a chronopotentiometric measurement was initiated at each specified current density and maintained for 100 s. Temperature data were collected during both the “heating” and “cooling” phases of the electrode by continuing the collection of emission spectra a few scans after the end of the chronopotentiometric measurement. Figure 2b shows a typical temperature curve obtained through luminescence thermometry, where the temperature increase at each data point is calculated using the equation:
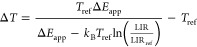
2where *T*_ref_ is the bulk temperature of the electrolyte (293 K, assumed to remain constant during the experiments) as measured by an external thermocouple and LIR_ref_ is the average luminescence intensity ratio of the first data points before applying a current. The derivation of eq 2 is given in the Supporting Information. The data points up to the end of the chronopotentiometric measurements (“heating”) were fitted to a logistic function, as given by
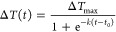
3where Δ*T*_max_ is the maximum value of the step (i.e., maximum temperature increase), *k* is the steepness of the curve, and *t*_0_ is the value of the midpoint of the S-shaped curve. The data points after the chronopotentiometric measurements could not be fitted by eq 3 and display exponential decay. The residuals of the fit, *i.e.*, fitting deviation, were used to calculate the error in the temperature increase after applying a current density (bottom of Figures 2b and Figure S3). Figure 2c illustrates the temperature changes observed at applied current densities ranging from −20 to −150 mA/cm^2^ and back to −20 mA/cm^2^ during HER, measured in a 0.1 M KOH electrolyte. The average temperature change (Δ*T*) demonstrates a direct correlation with the increasing current density and is fully reversible without hysteresis, reaching a maximum value of 2 K at −150 mA/cm^2^.

**Figure 2:**
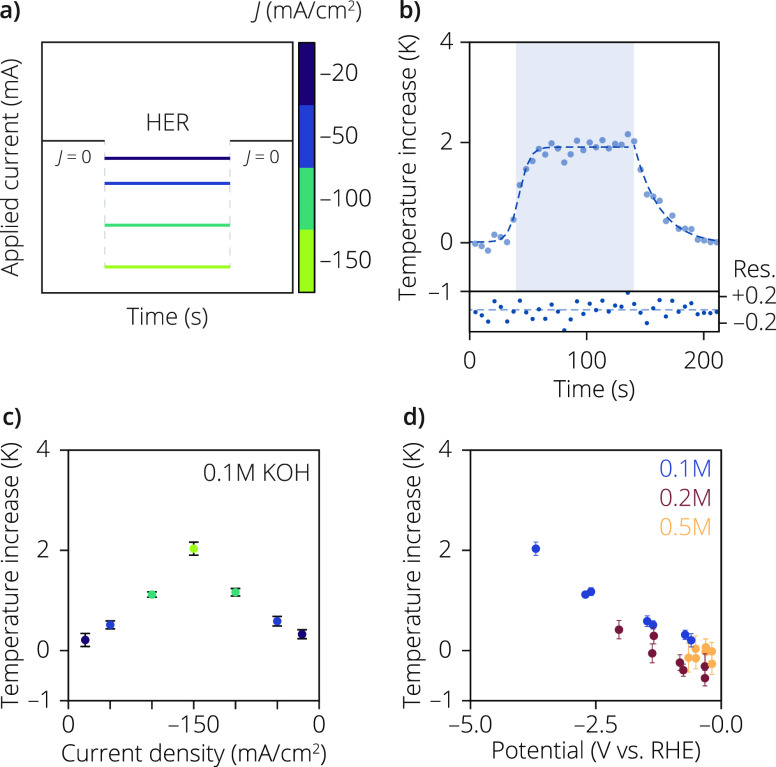
(a) Measurement procedure during the hydrogen evolution reaction (HER) on the bifunctional electrode. The temperature measurements were initiated at open circuit potential, and after a few measurements (around 30 s), the current density was set to values ranging from −20 to −150 mA/cm^2^ (from purple to light green, respectively) and the potential was measured. (b) Characteristic temperature profile obtained from the luminescence thermometry measurements. The time per measurement was 5 s, to ensure sufficient signal intensity. The temperature increase was calculated using eq 2, using the bulk electrolyte temperature of 20 °C. The points in the blue area were measured during an applied current density. The first part of the temperature curve was fitted to a sigmoidal function, while the decrease in temperature was fitted to exponential decay. The residuals of the first fit were used to calculate the standard deviation on the temperature readout (bottom). (c) Temperature increases measured using the sigmoidal fitting procedure of eq 3, in 0.1 M KOH at current densities ranging from −20 to −150 mA/cm^2^, going from purple to yellow, respectively (see Figure S3). (d) Temperature increases as a function of the different measured potentials during chronopotentiometry, where the blue, red, and orange dots are measured in 0.1, 0.2, and 0.5 M KOH, respectively.

The heat balance in an electrochemical system is governed by reversible and irreversible components.^25,26^ The reversible part, known as Peltier heat, is determined by changes in interfacial entropy and transport of ions to and from the interface during electrochemical reactions.^26^ This contribution can either absorb or release heat, depending on the direction of entropy change; for instance, in a gas-evolving reaction such as HER, it typically absorbs heat, thereby cooling the system. The irreversible components, on the other hand, invariably lead to heat production and include heat generated due to overpotential (the heat produced when the system deviates from thermodynamic equilibrium during current flow) and Joule heating (the heat produced due to current passing through a material with finite resistance). The observed temperature increase for all current densities indicates a predominant contribution from irreversible heat sources, as a cooling effect is expected from Peltier heat of the HER/OER but is not observed in the measurements. The expected heat contributions from the reversible and irreversible contributions are summarized in Table S3 of the Supporting Information, together with a short description of their determination (Supplementary Note 1).^27−29^ Another contribution to local heat could be a change in the valence state of the platinum electrode surface under oxidation conditions. However, we note that these heat effects are expected to occur at early times (few seconds) in the chronopotentiometry experiments, much shorter than the time scale of the luminescence thermometry measurements (∼100 s). Therefore, we refrained from discussing changes in the catalyst itself and the resulting heat effects in this study, as the platinum catalyst maintains a stable valence state, chemical composition, and crystal structure under the operating conditions during the temperature measurements.

In Figure 2d, Δ*T* values during HER are presented as a function of applied potential at varying electrolyte concentrations, ranging from 0.1 to 0.5 M KOH. Lower overpotentials are observed at higher electrolyte concentrations, attributed to increased ionic conductivity of the electrolyte. Notably, in 0.2 M KOH electrolyte solution, the Δ*T* values also increase with current density, although the values are notably lower than in 0.1 M KOH. This difference can be attributed to decreased overpotential heating and/or reduced Joule heating as the electrolyte concentration increases. As the electrolyte concentration is further increased to 0.5 M KOH, the measured Δ*T* is very small for all applied current densities that we measured. It also seems that there is almost no change in Δ*T* for the lower current densities at early times, whereas a slight increase in Δ*T* is observed over time (Figure S3). This could indicate the interfacial contributions (*i*.*e*., Peltier heat/cooling and overpotential heat) cancel each other out, and after a while, the Joule heat dissipates to the surface.

We also conducted temperature measurements at positive applied current densities to investigate the oxygen evolution reaction (OER) on the same Pt electrode. Figure 3a illustrates the measurement procedure, which mirrors the current density values used during the HER experiments. Due to the sluggish kinetics and OH^–^ consumption during the OER, the overpotential required for a given current density is notably higher compared to the HER (Figure S4). Unfortunately, the potential range of the potentiostat was not compliant for the measurements at current densities above +50 mA/cm^2^ in 0.1 M KOH, but it can be assumed that the applied potential would exceed the limit of 4 V. As a result, we focused solely on measurements conducted in electrolyte concentrations of 0.2 and 0.5 M KOH, which resulted in potentials below 4 V. Figure 3b presents the measurements in 0.2 M KOH up to +100 mA/cm^2^, where Δ*T* increases with a rising current density. Notably, in contrast to the case of HER, where similar changes in Δ*T* are observed, a more pronounced hysteresis in Δ*T* is observed during the OER: that is, the average temperature during the backward run is higher at the same applied current density, accompanied by a corresponding increase in the required potential during the second run to reach that current density during the OER (Figure S4), attributed to bubble fouling. As shown in chronopotentiometry graphs in Figure S4, during HER, a potential jump is observed due to the detachment of large H_2_ gas bubbles, while during the OER, no such potential jump is observed. This suggests that large O_2_ gas bubbles stay longer on the electrode surface compared to H_2_ gas bubbles,^30^ thus leading to a hysteresis of the measured potential during OER. Although examining detailed gas bubble detachment is beyond the scope of this study, we anticipate that the lower generation rate of O_2_ molecules compared to H_2_ molecules and the direction of the solutal Marangoni force are possible reasons for the longer detachment period of O_2_ gas bubbles under the same current conditions (Supplementary Note 2).^31,32^Figure 3c shows the results of the measurements in 0.5 M KOH, illustrating the same trends in temperature as we observed for 0.2 M KOH. Figure 3d illustrates that the overall Δ*T* in the 0.5 M KOH measurements is slightly lower compared to the 0.2 M KOH measurements, again indicating decreased overpotential heating and/or reduced Joule heating as the electrolyte concentration increases (Figures S8 and S9).^33^

**Figure 3:**
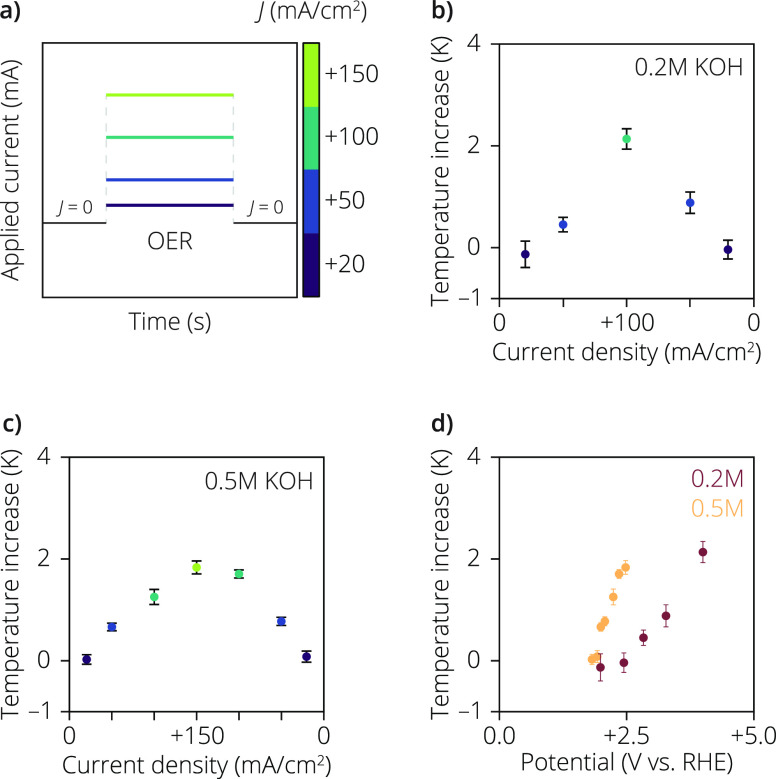
(a) Measurement procedure during the oxygen evolution reaction (OER) on the bifunctional electrode. The temperature measurements were initiated at open circuit potential, and after a few measurements (around 30 s), the current density was set to values ranging from +20 to +150 mA/cm^2^ (from purple to light green, respectively) and the potential was measured. (b) Temperature increases measured using the sigmoidal fitting procedure of eq 3, in 0.2 M KOH at current densities ranging from +20 to +100 mA/cm^2^, going from purple to dark green, respectively. The error bars are calculated based on the standard deviation of the residual of the sigmoidal fit in eq 3 (see Figure S5). (c) Same as in (b) but now in 0.5 M KOH (see Figure S6). As the overpotential of the reaction is lower at higher electrolyte concentrations, the temperature measurement could also be conducted at +150 mA/cm^2^. (d) Temperature increases determined at different measured potentials, where the red and orange dots are collected in 0.2 and 0.5 M KOH, respectively.

One key conclusion drawn from comparing Figures 2d and 3d is that the observed Δ*T* values are considerably higher during the OER than during the HER at identical electrolyte concentrations and applied current densities (Figure S7). Several potential factors could contribute to this observed phenomenon. First, the overpotential for OER is greater than that for HER, leading to increased overpotential heating. As the bulk conductivities of the electrode and electrolyte are the same in both cases, we expect a similar contribution from the heating of the bulk electrolyte. However, the Joule heating in the Nernst diffusion layer might vary between HER and the OER owing to several factors: (1) The local electrolyte conductivity decreases during the OER due to the depletion of OH^–^ ions, whereas it increases during the HER due to the generation of OH^–^ ions. (2) The electrode conductivity decreases due to the formation of Pt oxide at the surface during the OER. (3) The presence of O_2_ gas bubbles on the electrode surface for an extended period could increase the actual current density during the OER due to the fouling effect of the gas bubbles. While this paper does not quantify the exact impact of each factor, we note that identifying the most significant factor remains an essential direction for future research. For example, the effect of nonlinear transport phenomena (*e*.*g*., thermodiffusion and convection) on the observed interfacial temperature changes is still unknown. Using luminescence thermometry as a tool for these local temperature measurements, combined with the possibility of spatially resolved temperature measurements, could help in a better understanding and quantification of the factors governing the temperature dynamics at the electrode–electrolyte interface.

Luminescence thermometry is well suited to investigating local heat effects and temperature dynamics during both hydrogen and oxygen evolution reactions under alkaline conditions. It has shed light on the intricate interplay among current density, electrolyte concentration, and temperature changes at the electrode–electrolyte interface. Through time-resolved temperature measurements, we have observed a local current-dependent temperature increase up to 2 K. We attribute the temperature increase primarily to irreversible heat effects and reveal electrolyte-dependent variations in local heat effects during HER and OER. Our findings show the importance of electrolyte concentration in modulating overpotentials and subsequent temperature variations, with higher concentrations exhibiting more efficient ion transport and thus reduced polarization and Joule heating. By developing a bifunctional electrode that can measure the temperature at the electrode–electrolyte interface, we have gained insights into the temperature dynamics during electrochemical reactions, paving the way for an enhanced understanding and optimization of electrocatalytic processes. These findings not only contribute to fundamental knowledge in electrochemistry but also hold significant implications for the development of stable, efficient, and sustainable electrochemical energy conversion technologies.

## Supporting Information Available

Experimental methods, additional characterization, temperature curves, and calculations on the different heat generating factors (Peltier heat and Joule heating) (PDF)
