# Supplementary material for: Luminescence Thermometry Probes Local Heat Effects at the Platinum Electrode Surface during Alkaline Water Electrolysis
Source: ACS Energy Lett. 2024 Jun 13;9(7):3335–41. doi: 10.1021/acsenergylett.4c01238 (PMC11250089; doi:10.1021/acsenergylett.4c01238)
Supplement: Supplementary file 1 — nz4c01238_si_001.pdf [file nz4c01238_si_001.pdf]

Supporting Information for:

Luminescence Thermometry Probes Local Heat Effects at the Platinum Electrode Surface during Alkaline Water Electrolysis

AUTHOR NAMES

Thimo S. Jacobs [a),†], Sunghak Park [b,c,d),†], Marco Schönig [b)], Bert M. Weckhuysen [a)], Marc T.M. Koper [b),] and Ward van der Stam [a),*]*

AUTHOR ADDRESS

a) Inorganic Chemistry and Catalysis, Debye Institute for Nanomaterials Science & Institute for Sustainable and Circular Chemistry, Utrecht University, 3584 CG Utrecht, The Netherlands

b) Leiden Institute of Chemistry, Leiden University, 2300 RA Leiden, The Netherlands

c) Current address: Department of Future Energy Engineering, Sungkyunkwan University (SKKU), Suwon 16419, Republic of Korea

d) SKKU Institute of Energy Science and Technology (SIEST), Sungkyunkwan University, Suwon 16419, Republic of Korea

† Both authors contributed equally to this work.

*E-mail: w.vanderstam@uu.nl and m.koper@lic.leidenuniv.nl

Experimental methods

Chemicals and Materials

All chemicals were used without further purification. The chemicals Liquinox phosphate-free liquid detergent, acetone (99.5%), ethanol (99.8%), isopropanol (99.5%), methanol (99.8%), $Y(NO_3)_3 \cdot 6H_2O$ and $Nd(NO_3)_3 \cdot 6H_2O$ (both with a purity of 99.99%) were purchased from Sigma-Aldrich. Potassium hydroxide (85%, extra pure) was purchased from Acros Organics. Ammonium oxalate was purchased from J.T. Baker Chemicals. Ammonia (25%) was purchased from Merck. All chemicals used as received without further purification. Fluorine-doped tin oxide coated glass (FTO) with a surface resistivity of 7 Ω sq^{-1} was obtained (Sigma Aldrich) and cut into 1.0 cm × 1.0 cm pieces.

Luminescent thermometer synthesis

The microcrystalline Y_2O_3:Nd^{3+} was prepared via a precipitation technique based on the work of Buijs *et al.*[1] 4 mmol of the $RE(NO_3)_3$ (RE = Y and Nd) was dissolved in 10 mL ultra-pure water (MQ) and 12 mmol of ammonium oxalate was dissolved in 50 mL MQ. While stirring, the ammonium oxalate was added to the $RE(NO_3)_3$ solution causing RE oxalate to precipitate. The pH was checked using a pH indicator strip. Ammonia was added until the pH was around 9, after which the solution was stirred for 1 hour. Afterward, the solution was centrifuged (5000 rpm for 5 min), decanted and the precipitate was washed three times with water (MQ). The sample was dried overnight at 373 K and calcined at 1473 K in static air for 8 h using a ramp of 5 K/min.

Preparation of the bifunctional electrode

The FTO substrate was cleaned through sequential sonication with Liquinox detergent, acetone, ethanol, isopropanol, and deionized water. Subsequently, a dispersion of Y_2O_3 particles in methanol (10 mg mL^{-1}) was prepared, and 10 µL of the dispersion solution was drop-coated onto the FTO substrate over an area of 0.5 cm^2 after masking the substrate. A 100 nm thick layer of Pt was then deposited using a sputter coater (sputter coater 208HRD, Cressington) equipped with a Pt target (99.9%, chemPUR) over the entire Y_2O_3/FTO sample. To improve adhesion to the substrate, the sample underwent heat treatment at 550 °C in air atmosphere. Additional extension for electrical connection was made with Ti wire (99.8%, MaTeck) together with Ag conductive paint (AGG3691, agar scientific). The Ag conductive paint was covered by an insulating hot-melt adhesive to prevent exposure to the electrolyte.

Materials Characterization

The surface morphology of the prepared sample was analyzed using Scanning Electron Microscopy (SEM, Apreo, Thermo Scientific). SEM images were obtained with a beam condition of 15 kV and 0.1 nA using an Everhart-Thornley detector. The controlled heating for the calibration of the thermometer was performed in a Linkam TS1200 microscope stage without a gas flow. X-ray diffraction (XRD) pattern of $Y_2O_3:Nd^{3+}$ was recorded with a Bruker D2 Phaser using Co Kα (λ = 1.79026Å) radiation (Figure S1). The measured range in terms of 2θ was 20–80° with a step size of 0.02°. Inductively Coupled Plasma Optical Emission Spectroscopy (ICP-OES) was performed on a PerkinElmer ICP-OES Optima 8300 to determine the dopant concentration of Nd^{3+} in Y_2O_3. The neodymium lines used were 401.225 and 406.109 nm and the results from these lines were averaged. The yttrium lines used were 324.227 and 371.029 nm and the results from these lines were averaged. The amount of Nd^{3+} in the Y_2O_3 crystal lattice was calculated to be 1.15% ± 0.11.

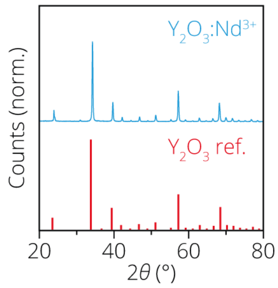

Figure S1. X-Ray Diffraction pattern of $Y_2O_3:Nd^{3+}$, matching the reference (red bars, database no. 00-041-1105).

Temperature measurements during water electrolysis

The neodymium emission spectra were measured using a Horiba Raman Spectrometer with the 785 nm laser, 50× objective (NA = 0.5), and 1% laser power (0.48 mW or 10^5 W/cm^2). The laser power used does not cause illumination-induced heating of the sample.[2] All emission spectra were corrected for a spectrum that was collected with the same measurement duration, but without laser. All electrochemical experiments were performed in a solution of KOH in milli-Q water without gas purging. A platinum wire (99.9%, MaTeck) and a reversible hydrogen electrode (RHE HydroFlex, Gaskatel) were used as counter electrode and reference electrode, respectively. The platinum wire was annealed before placing it in the electrolyte solution. The cyclic voltammetry and chronopotentiometric (CP) were conducted using an Ivium CompactStat.h10800 potentiostat. Solution resistance was determined from the impedance measurement (Figure S8) and compensated manually afterward to calculate overpotential values for each condition (Figure S9).

Supplementary Note 1. Evaluation of different heat contributions during electrolysis

As pointed out in the main text, the heat exchanged during electrochemical processes consists of reversible and irreversible contributions.[3] In the following part, we will give a short description of the origin of each heat contribution and explain how they can be calculated.

Reversible Peltier Heat

During an electrochemical reaction, heat contributions arise from the ongoing reaction and the corresponding entropy change $\Delta_R S$, as well as from the ion transport from and to the interface $\Delta_T S$.[4] The molar Peltier heat, i.e. the heat change upon the conversion of one mole of electrons, can be calculated in the following way:

$$\Pi = T(\Delta_R S - \Delta_T S) \quad \textbf{(S1)}$$

The reaction entropy can be calculated from the partial molar entropy s_i of each species involved in the reaction by:

$$\Delta_R S = \sum_{products} s_i - \sum_{reactants} s_i \quad \textbf{(S2)}$$

The absolute partial molar entropies of all species involved in the HER and OER are summarized in Table S1. We corrected the deviation from standard conditions by assuming that the activity coefficient is approximately 1, *i.e.*, using the concentrations of the employed solutions.

Table S1: Absolute molar entropies of the products and reactants involved in the HER and OER.

	H_2	O_2	OH^-	H_2O
s_i^0 (J mol K $^{-1}$)	130.7[5]	205.2[5]	11.4[6]	70[7]

The entropy change due to transport can be calculated from the Eastman entropies of transport $\hat{s}_i$ of each ion i and its Hittorf transference number t_i by:

$$\Delta_T S = \sum_i \frac{t_i \hat{s}_i}{z_i} \quad \textbf{(S3)}$$

The Hittorf transference numbers were calculated using the limiting ionic conductivities[8] and the Eastman entropies of transport were taken from Ref 4. Under the approximation that the activity coefficient is close to 1, there is no difference between the different concentrations. The obtained values are summarized in Table S2, together with the resulting transport entropy.

Table S2: Values for Eastman entropies of transport ($\hat{s}_i$), Hittorf transference number (t_i) and the entropy change ($\Delta_T S$), following from Eq. 3.

	$\hat{s}_i$ (J mol K^{-1})		t_i		$\Delta_T S$ (J mol K^{-1})
	K^+	OH^-	K^+	OH^-	
KOH	4	53.4	0.271	0.729	-37.8

The water reduction reaction in alkaline electrolytes can be written as:

$$2H_2O + 2e^- \rightarrow H_2 + 2OH^- \quad \textbf{(S4)}$$

Using the values from Table S1 in Eq. S2, this results in $\Delta_R S$ = 51.8 J mol_{H2}^{-1} K^{-1}, resp. 25.9 J mol_{e-}^{-1} K^{-1} in 0.1 M KOH. Thus, the resulting Peltier heat resulting from Eq. S1 amounts to 19.1 kJ mol^{-1}. Using a current density of –100 mA cm^{-2}, which means a current flow of 50 mA in our setup, we expect a cooling of $\Pi/_F \cdot I$ = –0.01 J s^{-1} during the HER in 0.1 M KOH.

Similarly, the Peltier heat during water oxidation reaction can be calculated as follows:

$$4OH^- \rightarrow O_2 + 2H_2O + 4e^- \quad \textbf{(S5)}$$

The reaction entropy of this reaction amounts to 55.7 J mol_{e-}^{-1} K^{-1} and a Peltier heat of 26.7 kJ mol^{-1} (please note that the Peltier heat is defined in the cathodic direction and therefore its value must be inversed for the calculation of the heat flow during the reaction). For a current flow of +50 mA, we expect a cooling of –0.015 J s^{-1} in 0.1 M KOH. The results for the different employed solutions are summarized in Table S3.

Polarization heat

As pointed out by for example Ref. 9 and Ref. 10, the application of an overpotential to drive the reaction leads to an irreversible heating effect that is typically generated in the vicinity of the electrode.[9,10] The heat generated by the applied overpotential can be calculated via:

$$\Phi_{pol} = |\eta|\, I \quad \textbf{(S6)}$$

The IR–corrected overpotential reached during the CP measurement at –50 mA in 0.1 M KOH amounts to around –0.13 V. Using Eq. S6 this yields an expected heat flux of ϕ_{pol}=0.065 J s^{-1}. The results for the different employed solutions are summarized in Table S3.

Joule heating

Joule heating is the heating caused by interaction between the charge carriers and the conducting medium and typically scales linearly with the resistance. In the case of an electrolyte solution this is typically caused by the movement of ions and resulting enthalpic effects like Stokes friction. For the bulk electrolyte it can be calculated by:

$$\Phi_{\mathrm{Joule}} = R\, I^2 \tag{S7}$$

For an 0.1 M KOH solution with a resistance of 51.5 Ω this would result in a heating of 0.13 J s^{-1} (see Figure S8a). It has to be noted that compared to the other two heat sources that are generated directly at the interface, the Joule heat is produced along the whole current path. As the Y_2O_3 particles detect the temperature change at the electrode, we expect only a fraction of the Joule heat to be dissipated in the direction of the surface, while most of the heat will be consumed to heat the whole electrolyte volume.

Summary of the three considered heat contributions

Table S3: The results for the different employed solutions (Peltier, polarization and Joule heating) in the studied electrolyte concentrations at the specific current densities.

		Φ_{Peltier}		Φ_{pol}		Φ_{Joule}
		HER	OER	HER	OER	
0.1 M KOH	20 mA cm^{-2}	–0.002	–0.003	0.001		0.005
	50 mA cm^{-2}	–0.005	–0.007	0.002		0.032
	100 mA cm^{-2}	–0.010	–0.015	0.007		0.129
	150 mA cm^{-2}	–0.015	–0.022			0.290
0.2 M KOH	20 mA cm^{-2}	–0.002	–0.003	0.001	0.006	0.002
	50 mA cm^{-2}	–0.005	–0.008	0.007	0.028	0.012
	100 mA cm^{-2}	–0.009	–0.015	0.020	0.092	0.047
	150 mA cm^{-2}	–0.014	–0.023	0.047		0.106
0.5 M KOH	20 mA cm^{-2}	–0.002	–0.003	0.001	0.005	0.001
	50 mA cm^{-2}	–0.004	–0.008	0.003	0.015	0.004
	100 mA cm^{-2}	–0.008	–0.017	0.008	0.034	0.016
	150 mA cm^{-2}	–0.012	–0.025		0.057	0.04

Not considered heat contributions

In comparison to the isothermal and close-to-equilibrium conditions considered in the derivation of the Peltier heat and overpotential heat, under the conditions employed in this study, considerable heat and concentration gradients are expected. This will lead to non-linear transport effects, e.g. thermodiffusion, which are not considered in the above discussion. This might also lead to a derivation of the origin of the overpotential heat from only the interface, as the Nernst diffusion layer build up during electrolysis will span further into the bulk electrolyte and its relaxation will take up several minutes. Further, the extensive bubble formation will lead to convection effect and thus an additional heat contribution, that also affects the ion and heat transport.

Supplementary Note 2. Possible origins of different gas bubble fouling effects between H_2 and O_2 gas bubbles

The fouling effect due to the gas bubbles covering on the electrode surface is directly influenced by how long gas bubbles stay on the electrode surface, i.e. gas bubble detachment period. This detachment period can be approximately estimated using Faraday's law and ideal gas law:

$$\text{detachment period} = \left|\frac{n}{r}\right| \cong \left|\frac{PV/RT}{I/zF}\right| = \left|\frac{zFPV}{RTI}\right| \qquad \textbf{(S8)}$$

where n, r, I, z, F, P, V, R, and T represent the number of gas molecules in the gas bubble at the detachment, the generation rate of gas molecules from the electrode, the current, the number of electrons required to produce a gas molecule (2 for H_2 and 4 for O_2), the Faraday constant, the pressure, the volume of the gas bubble, the gas constant, and the temperature, respectively. Here we assume that all produced gas molecules are captured by gas bubbles. Since a gas bubble detaches when its size is sufficient to overcome other forces, the buoyancy force (F_{buo}) should equal the sum of other forces (F_{other}) at the moment of detachment, and hence the gas bubble detachment volume can be further expressed by the following equations:

$$V = \left|\frac{F_{\mathrm{buo}}}{\rho g}\right| = \left|\frac{F_{\mathrm{other}}}{\rho g}\right|, \qquad \textbf{(S9)}$$

where ρ is the electrolyte's density and g is gravitational acceleration. Therefore, the detachment period is:

$$\text{detachment period} \cong \left|\frac{zFPF_{\mathrm{other}}}{RTI\rho g}\right| \qquad \textbf{(S10)}$$

Under identical electrochemical conditions (particularly the same current), the detachment period is influenced by z and F_{other}. One possible reason for the observed longer residence time of O_2 gas bubble is likely the larger z for O_2 ($z = 2$ for HER and $z = 4$ for OER). In other words, the smaller amount of generated O_2 gas molecules leads to slower growth, contributing to a longer detachment period. Additionally, the difference in F_{other} between H_2 and O_2 gas bubbles plays a role. Although it is challenging to precisely quantify all other contributing forces, the solutal Marangoni force, known for a critical factor affecting single H_2 gas bubble detachment,[11,12] significantly differs between H_2 and O_2 gas bubbles. In alkaline solution, the solutal Marangoni force for O_2 gas bubble would be directed towards the electrode due to reduced surface tension near the electrode region during OER, while for H_2 gas bubbles, it is directed away from the electrode due to increased surface tension near the electrode region during HER. Thus, a larger F_{other} for O_2 gas bubbles is expected, which contributes to a longer detachment period.

Characterization of the bifunctional electrode with SEM

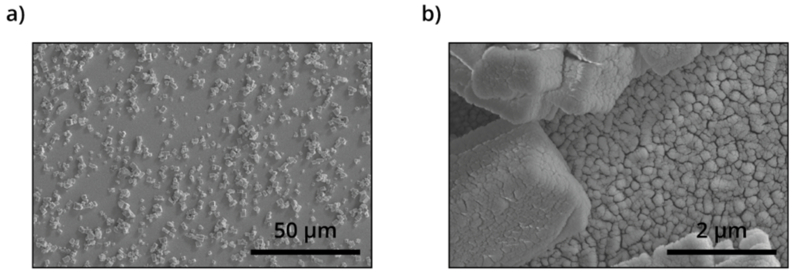

Figure S2. a) Scanning Electron Microscopy (SEM) image of the surface of the electrode, where the larger cubic particles are the thermometry particles. The scalebar is 50 µm. b) Same as in a), but zoomed-in to show the sputtered layer of platinum particles on top of the Y_2O_3 thermometry particles. The scalebar is 2 µm.

Derivation of equation to calculate Δ*T*

We used the change in LIR to calculate the temperature increase, assuming that the local scattering and/or absorption and therefore the prefactor *C* were the same (which holds at the same pixel). Eq. S11 was derived from Eq. 1 in the main text:

$$\frac{\mathrm{LIR}}{\mathrm{LIR}_{\mathrm{ref}}} = \exp\left(-\frac{\Delta E_{\mathrm{app}}}{k_{\mathrm{B}}T} + \frac{\Delta E_{\mathrm{app}}}{k_{\mathrm{B}}T_{\mathrm{ref}}}\right) \quad \textbf{(S11)}$$

$$T = \frac{T_{\mathrm{ref}}\Delta E_{\mathrm{app}}}{\Delta E_{\mathrm{app}} - k_{\mathrm{B}}T_{\mathrm{ref}}\ln\left(\frac{\mathrm{LIR}}{\mathrm{LIR}_{\mathrm{ref}}}\right)} \quad \textbf{(S12)}$$

Fits + residuals of HER in 0.1M KOH

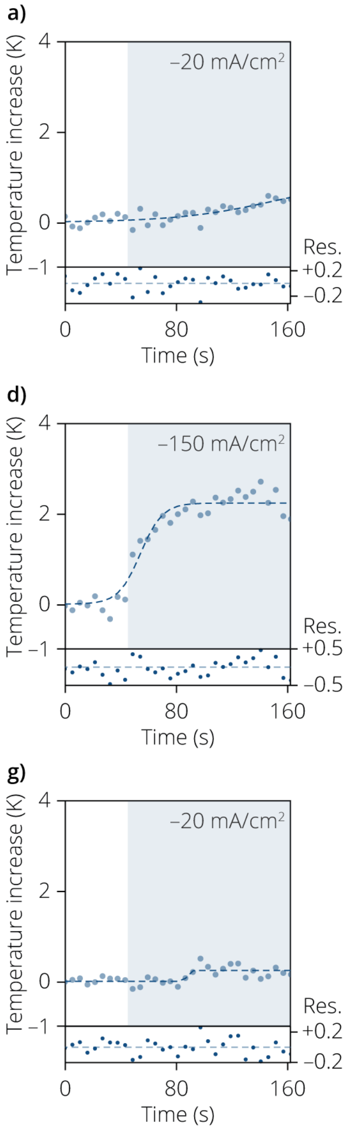

Figure S3. Temperature measurements at different current densities in 0.1M of KOH during HER. Panels a–d) are at increasing current densities from –20 to –150 mA/cm^2, while panels e–g) show the reversibility measurements at decreasing values back to –20 mA/cm^2. The dotted line in all temperature graphs is the sigmoidal fit, where the blue rectangle is placed at the datapoints where the potentiostat was applying a current. The dotted line in all residual plots is 0.

Chronopotentiometric measurements

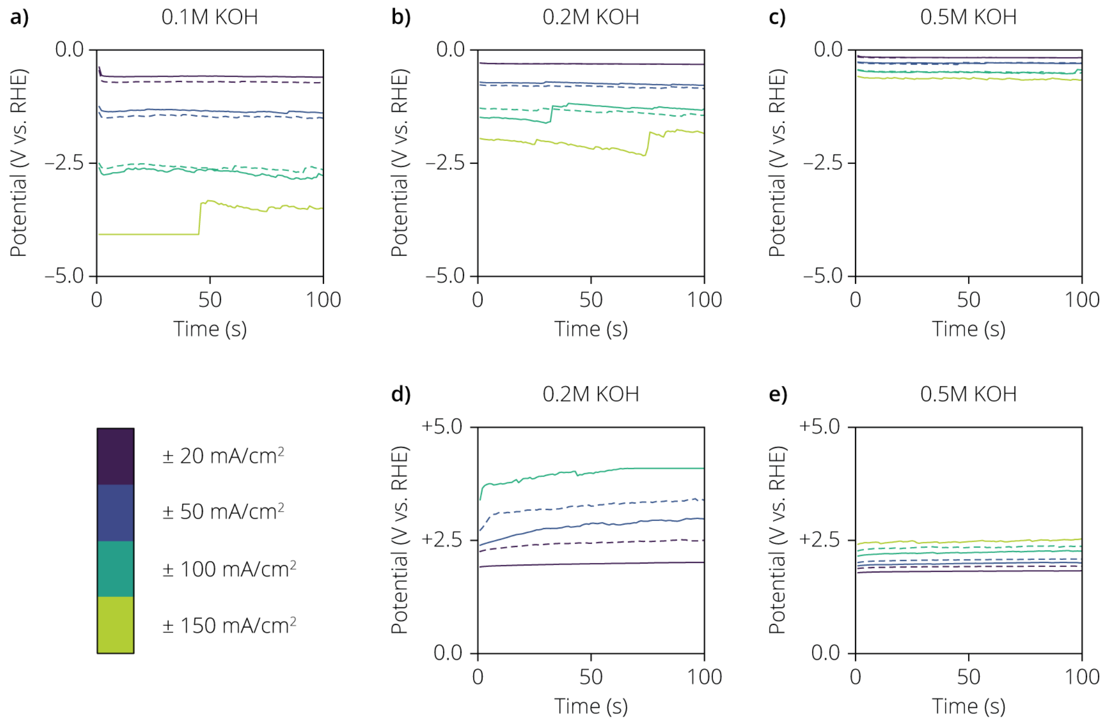

Figure S4. Chronopotentiometric (CP) measurements at the different electrolyte concentrations. The measurements in panels a–c) are all performed in HER conditions, varying the KOH concentration from 0.1M to 0.2M and 0.5M, respectively. Panels d) and e) show the measurements in OER conditions, with KOH concentrations of 0.2M and 0.5M, respectively. The light green line in panel a) and the darker green line in panel d) both show the limits of the potentiostat, but the desired current density was still achieved.

Fits + residuals of OER in 0.2M KOH

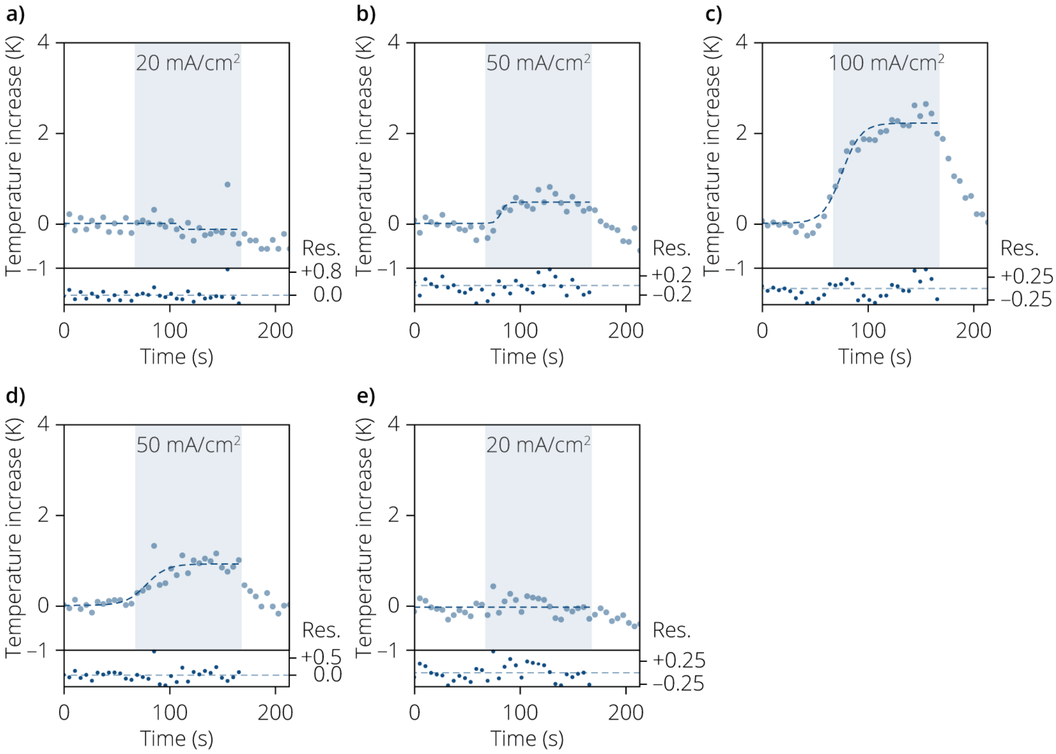

Figure S5. Temperature measurements at different current densities in 0.2M of KOH during OER. Panels a–c) are at increasing densities from 20 to 100 mA/cm^2, while panels d,e) show the reversibility measurements at decreasing values back to 20 mA/cm^2. The dotted line in all temperature graphs is the sigmoidal fit, where the blue rectangle is placed at the datapoints where the potentiostat was applying a current. The dotted line in all residual plots is 0.

Fits + residuals of OER in 0.5M KOH

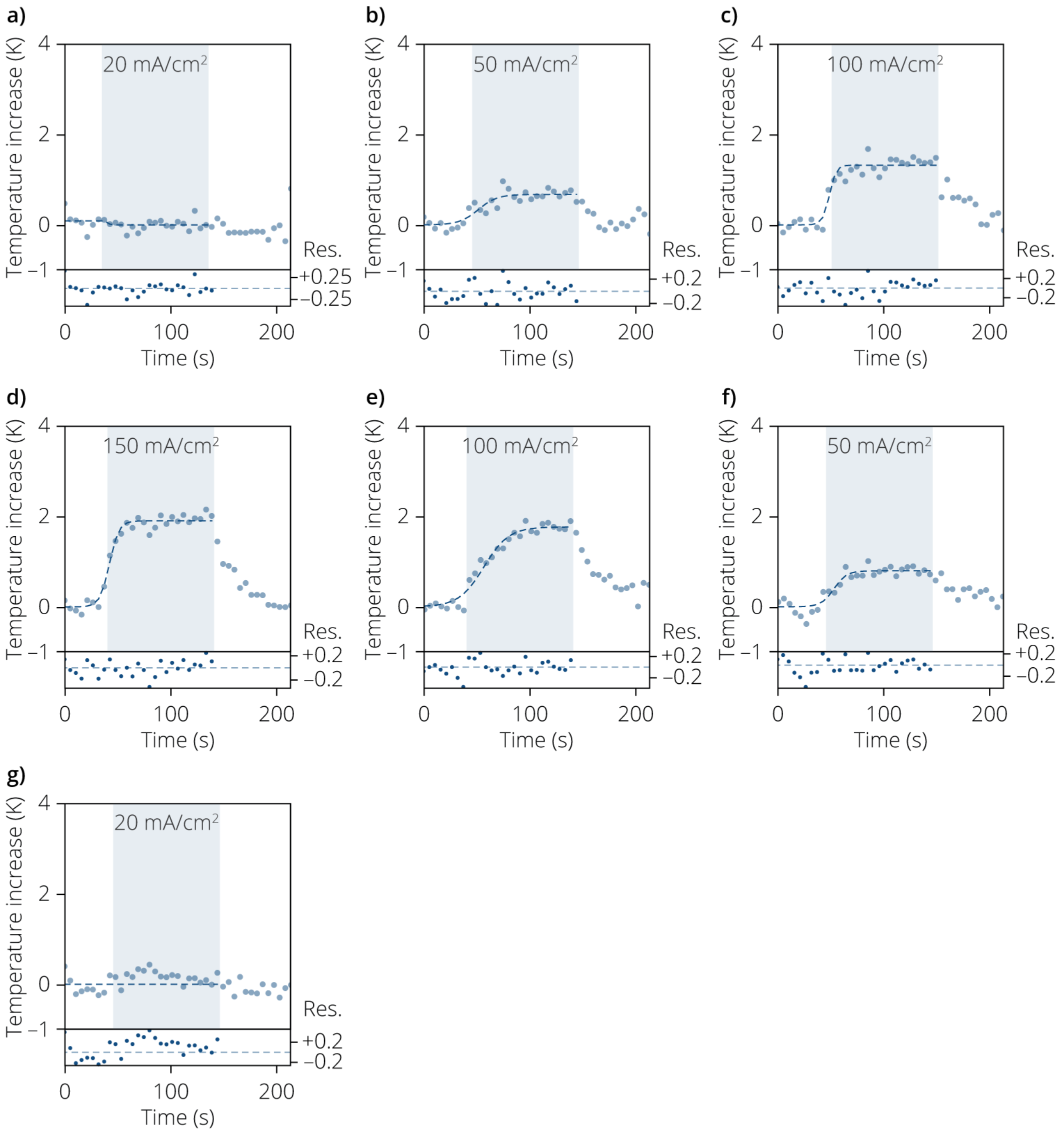

Figure S6. Temperature measurements at different current densities in 0.5M of KOH during OER. Panels a–d) are at increasing densities from 20 to 150 mA/cm^2, while panels e–g) show the reversibility measurements at decreasing values back to 20 mA/cm^2. The dotted line in all temperature graphs is the sigmoidal fit, where the blue rectangle is placed at the datapoints where the potentiostat was applying a current. The dotted line in all residual plots is 0.

Comparison of ΔT for the OER and HER

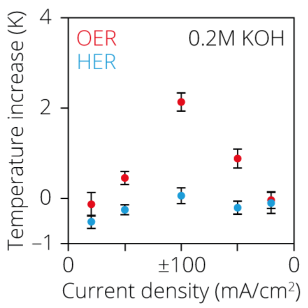

Figure S7. Comparison of observed ΔT values for the OER (red) and HER (blue) at identical electrolyte concentration (0.2M KOH) and current densities).

Impedance measurements

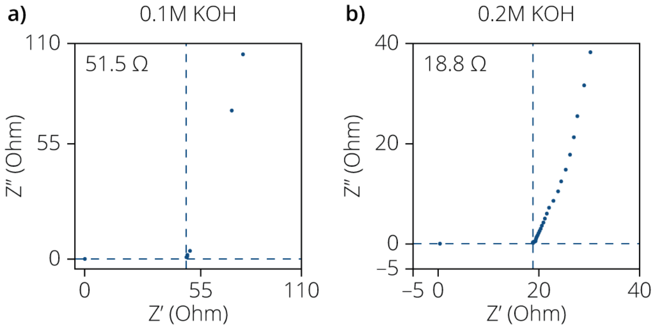

Figure S8. Impedance measurements in a) 0.1M KOH and b) 0.2M KOH. The solution resistivity for 0.5M KOH was estimated by linear extrapolation of the solution resistivities in the other electrolyte concentrations.[13]

iR corrected plots of ΔT vs. overpotential

The iR correction was performed by multiplying the current (mA) with the respective solution resistance (Figure S8) for the different electrolyte concentrations. Note that here we employed constant solution resistance values, measured at open-circuit potential, across different current density conditions. This approach indicates that our analysis did not account for the potential fouling effects induced by gas bubble formation, which could alter the resistance, especially under high current density conditions where gas bubble evolution is vigorous.

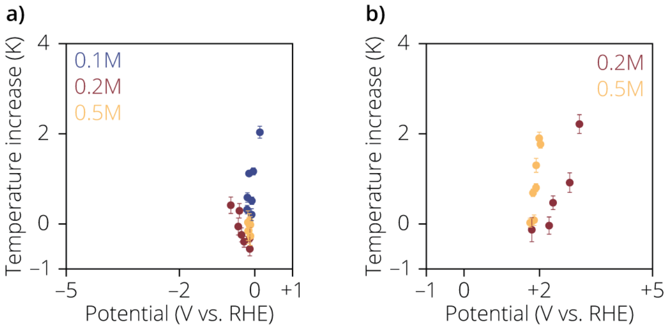

Figure S9. a) Temperature increases measured during HER at iR corrected potentials, where the blue, red and orange dots are measured in 0.1M, 0.2M and 0.5M KOH, respectively. b) Same as in a), but now for the OER.

References

1. Buijs, M., Meyerink, A. & Blasse, G. Energy Transfer between Eu^{3+} Ions in a Lattice with Two Different Crystallographic Sites: $Y_2O_3:Eu^{3+}$, $Gd_2O_3:Eu^{3+}$ and Eu_2O_3. *J. Lumin.* **37**, 9–20 (1987).
2. Jacobs, T. S. *et al.* Mapping Temperature Heterogeneities with Micrometer Resolution during Catalytic CO_2 Methanation with Operando Luminescence Thermometry. *ACS Nano* **17**, 20053–20061 (2023).
3. Schuster, R. Electrochemical microcalorimetry at single electrodes. *Curr. Opin. Electrochem.* **1**, 88–94 (2017).
4. Agar, J. N. Thermogalvanic cells. in *Advances in Electrochemistry and Electrochemical Engineering* (eds. Delahay, P. & Tobias, C. W.) 31–121 (Interscience, 1963).
5. Huber, K. P. & Herzberg, G. H. Constants of Diatomic Molecules. in *NIST Chemistry WebBook, NIST Standard Reference Database Number 69* (eds. Linstrom, P. J. & Mallard, W. G.) (2024).
6. Marcus, Y. *Ion Solvation*. (Wiley, 1985).
7. Conway, B. E., Angerstein-Kozlowska, H. & Sharp, W. B. A. Temperature and pressure effects on surface processes at noble metal electrodes. Part 1. - Entropy of chemisorption of H at Pt surfaces. *J. Chem. Soc. Faraday Trans. 1 Phys. Chem. Condens. Phases* **74**, 1373–1389 (1978).
8. Lide, D. R. *CRC Handbook of Chemistry and Physics*. (CRC Press, 1990).
9. Kuz'minskii, Y. V. & Gorodyskii, A. V. Thermal analysis of electrochemical reactions. Part I. Kinetic method of determining Peltier heats. *J. Electroanal. Chem.* **252**, 21–37 (1988).
10. Bickel, K. R., Etzel, K. D., Halka, V. & Schuster, R. Microcalorimetric determination of heat changes caused by overpotential upon electrochemical Ag bulk deposition. *Electrochim. Acta* **112**, 801–812 (2013).
11. Park, S. *et al.* Solutal Marangoni effect determines bubble dynamics during electrocatalytic hydrogen evolution. *Nat. Chem.* **15**, 1532–1540 (2023).
12. Park, S., Lohse, D., Krug, D. & Koper, M. T. M. Electrolyte design for the manipulation of gas bubble detachment during hydrogen evolution reaction. *Electrochim. Acta* **485**, 144084 (2024).
13. Gilliam, R. J., Graydon, J. W., Kirk, D. W. & Thorpe, S. J. A review of specific conductivities of potassium hydroxide solutions for various concentrations and temperatures. *Int. J. Hydrogen Energy* **32**, 359–364 (2007).
